# Disaster health education framework for short and intermediate training in Saudi Arabia: A scoping review

**DOI:** 10.3389/fpubh.2022.932597

**Published:** 2022-07-29

**Authors:** Nidaa Bajow, Luc J. M. Mortelmans, Nisreen Maghraby, Salem Ali Alatef Sultan, Zakaria A. Mani, Samer Aloraifi

**Affiliations:** ^1^Unit of Disaster Medicine, Emergency Department, Security Force Hospital Programs, Riyadh, Saudi Arabia; ^2^Department of Emergency Medicine, Ziekenhuisnetwerk Antwerpen (ZNA) Camp Stuivenberg, Center for Research and Education in Emergency Care (CREEC) Univ Louvain Belgium and Regedim Free University, Brussels, Belgium; ^3^Emergency Medicine Department, College of Medicine, Imam Abdulrahman Bin Faisal University, Dammam, Saudi Arabia; ^4^Surgery Department, King Fahad Hospital, Najran, Saudi Arabia; ^5^School of Nursing and Midwifery, Monash University, Frankston, VIC, Australia; ^6^College of Nursing, Jazan University, Jazan, Saudi Arabia; ^7^Surgery Department, Doctor Sulaiman Alhabib Ar Rayyan Hospital, Riyadh, Saudi Arabia

**Keywords:** competency-based, disaster health, multidisciplinary approach, disaster curriculum, education framework

## Abstract

**Background:**

Saudi Arabia has made extensive efforts to manage disasters using unique national approaches; however, challenges and obstacles concerning disaster health handling persist. The nation has a reactive strategy to disaster management with a need for increased involvement of health professionals in disaster management and improvement of healthcare facilities emergency preparedness including competency-based education training.

**Objective:**

A comprehensive and consistent approach of disaster education programs for short and intermediate training of health professionals involved in disaster responses in Saudi Arabia is still not evident. Therefore, it is vital to explore and map the current state of the disaster education framework in Saudi Arabia.

**Methods:**

The Joanna Briggs Institute approach for scoping reviews was used to assess research articles and preprints between January 2000 and September 2021 from Saudi Digital Library; PubMed, CINAHL, and Google Scholar. Five experts identified key aspects of the disaster education approach and eligibility criteria to facilitate identification of relevant articles.

**Results:**

Only five articles met the specified criteria and described two short and three intermediate courses on disaster health management in Saudi Arabia. All courses involved competency-specific training aimed at basic or foundational level and involved a range of activities and learning types. None had refresher courses within 12 months.

**Conclusion:**

The review highlights the obvious scarcity of short and intermediate term evidence-based disaster health programs in Saudi Arabia. Adoption of the education framework proposed by the authors based on international frameworks could improve the quality and consistency of the disaster education curriculum in Saudi Arabia.

## Introduction

The magnitude and impact of disasters in Saudi Arabia have increased in recent years due to population overcrowding, terrorist attacks, technological incidents, climate changes, and social events. Floods have been the most important natural disaster in Saudi Arabia. Mass gathering during Hajj remain an important vulnerable event that creates a burden on public health emergencies and, infectious disease control. The threats of unique pathogens, such as the Middle East respiratory syndrome (MERS) was also faced. Moreover, political unrest in the Middle East worsens the possibility of catastrophe due to violent conflicts and terrorist attacks (1–4).

Saudi Arabia has made extensive efforts to manage disasters using unique national approaches; however, challenges and obstacles concerning disaster health handling persist. The nation has a reactive strategy to disaster management with a need for increased involvement of health professionals in disaster management and improvement of healthcare facilities emergency preparedness including competency-based education training (5–9). Disaster health education is the keystone for disaster preparedness. The poor performance and responses of healthcare systems to disaster are the result of inadequate or ineffective disaster health education and training (10). Health personnel empowerment *via* disaster health education based on gaining task-related, profession-specific, and cross-disciplinary competencies through accredited education programs is required to improve responses to disaster events by the Saudi healthcare system (11).

Previous research has demonstrated major obstacles to providing high-quality disaster health education programs in Saudi Arabia. This includes, but is not limited to, lack of formal disaster health education for both undergraduates and postgraduates; scarcity of competency-based education programs; the paucity of available expert educators; limited opportunities for training; limited financial support for disaster training from the workplace; and a dearth of disaster medicine research in the country (1, 12–15).

Most disaster medicine education research in Saudi Arabia focuses on the need for assessment and evaluation of knowledge and skills for healthcare workers: there is very limited research on evidence-based disaster health education programs in Saudi Arabia (1, 4, 12–21). Disaster health management is a multi-disciplinary body of knowledge with three primary overlapping domains, i.e., emergency medicine, public health, and disaster management. These domains provide a wide range of skill sets to comprehensively prepare for and approach disasters from a health perspective (22, 23). As a result, it is critical to focus on the collaborative application of various health disciplines of prevention, preparedness for, response to, and recovery from the health problems arising from a disaster.

The requisite preparedness levels can be achieved after witnessing several disaster scenarios or undergoing high-quality training. Acceptable preparedness involves the educational and physical resources of a nation including available facilities, finances, and requirements concerning outcomes and quality. Therefore, educational initiatives (EITs) are critical for offering disaster-related education using a comprehensive disaster health management framework (24).

Standardizing disaster health information and training might cause versatility issues due to organizational dynamics (24). However, standardization is expected to provide quality and consistency of education, enhance international efforts, and facilitate continuous assessment and enhancement. Inadequate standardization for handling disaster health effects is a critical roadblock inhibiting the effectiveness of such educational initiatives. Hence, Saudi Arabia must contemplate setting basic standards and measurement tools to assess disaster health education initiatives (16). The objective of this study was to explore and map the current state of disaster health education frameworks for short and intermediate training programs in Saudi Arabia based on a review of the relevant literature.

## Methods

The scoping review approach developed by academics from the Joanna Briggs Institute (JBI; Adelaide, Australia) was employed for the study. It comprises study objective identification, exclusion and inclusion constraints, determining a search approach, and obtaining and plotting the outcomes (25, 26). This approach comprises a step-wise process formulated for creating and mapping concerns related to the present literature associated with an area of interest (26, 27). In addition, the Preferred Reporting Items for Scoping Reviews (PRISMA-ScR) checklist was followed (27).

### Inclusion and exclusion criteria

This review involved original research articles and preprints (a preliminary article that has undergone peer review) related to disaster health education frameworks for short or intermediate training in Saudi Arabia. The Population, Concept, and Context (PCC) for scoping reviews was followed (26, 27). The population was the health community; the concept was two or more elements of the disaster health education framework according to international standards and local context identified by five experts (1, 11, 23, 28–32); and the context included healthcare facilities, organizations, and schools of medicine, health sciences, and emergency medical services (EMS) in Saudi Arabia.

#### Types of studies

Study inclusion criteria were applied to primary research work and full-text preprints from peer-reviewed journals published in English between January 2000 and September 2021. Studies fulfilling these inclusion requirements were collected for the review process.

#### Types of population

The target population included articles aimed at health personnel associated with healthcare institutions from practical or education perspectives, and individual experts from academic institutions such as hospital receivers, first medical responders, and experts on disaster medicine. Moreover, personnel with secondary responsibilities (e.g., healthcare providers, undergraduates, and academic directors for medical health) concerning disaster medicine and responses were also included. In this case, healthcare providers comprise professionals such as, nurses, technical staff, and specialty doctors as opposed to frontline responders. Home and rehabilitation nurses were not included.

#### Concepts

Articles that focused on disaster health education programs for short or intermediate training (<4 months) with two or more key elements of the disaster health education framework identified by the experts were included in the review. The term disaster health is used when considering the need for multi-disciplinary health response to disastrous incidents. Articles focused on simulations and drills without education intervention, or long-duration programs (>4 months) were excluded from the review.

#### Disaster health education framework

A disaster health education framework for short and intermediate training in Saudi Arabia was proposed by five disaster medicine experts (1, 11, 23, 28–32). The framework was based on international disaster health education frameworks and the roles of health personnel during disaster preparedness including response (either primary roles as frontline healthcare responders or secondary roles as healthcare providers and undergraduate medical students), education level, professional type, and organization roles. In this study, the framework was used as a benchmark to assess the representation of key elements of disaster health education in the articles describing short and intermediate training in Saudi Arabia.

The framework ensures health professionals are represented in each category with realization of the multiplicity of expected job functions and educational requirements for each health professional involved in disaster health preparedness including response. At the health personnel awareness level, the competency set is basic for all healthcare workers with secondary roles while at the foundation level, more advanced or specific competencies are essential. The health personnel level will need to be involved in refresher courses if their roles remain secondary. For the intermediate level (professional level) the competency set has more extensive knowledge and advanced skills and is cross-referenced for each professional group (paramedics, nurses, physicians in intensive care units and operating rooms and emergency departments). At the organization-level the competency set is highly specialized and integrated (1, 11, 23, 28–32).

##### Main elements in the disaster health education framework

**Part I: Level of training**

The criteria for classifying competency sets according to the proposed disaster health education framework (1, 11, 23, 28–32) are presented in Table 1.

**Table 1:** Criteria for classifying competency sets according to the proposed disaster health education framework.

**Level**	**Description**
Health personnel awareness level (secondary role)	- Health professionals with a secondary role in disaster preparedness and response who may be involved in disaster health responses, examples: - Healthcare providers - Undergraduate students in schools of Health Science, Medicine, Nursing and Emergency Medical Services. - The competency set is basic for all health personnel.
Foundation level (primary role)	- All front-line providers (first responders and hospital receivers), who are likely to be involved in a primary role during disaster health management. - Competency set is essential for more advanced / specific competencies (professional and organization levels).
Intermediate level (professional level) (primary role)	- Health professionals who play a significant role during disaster health responses. - The competency set includes more extensive knowledge and advanced skills with cross referencing for each professional group including paramedics, nurses, and physicians in EM & ICU and Trauma.
Advanced level (organization level) (primary role)	- Health professionals who have significant roles according to their organization (hospital, NGO, and EMS). - The competency set is highly specialized in integrated operation, strategy, and tactical planning.
Master	Not included
Expert/specialist-2 years of experience	Not included
PhD	Not included

**Part II: Other disaster health elements**

The other elements for the proposed disaster health framework (1, 11, 23, 28–32) are shown in Table 2.

**Table 2:** Elements for disaster health framework.

**Element**	**Explanation**
Multidisciplinary/multi-sector and inter professional approach	A multidisciplinary/multi-sector approach is a vital component of a disaster health education framework. The participants will be aware of the roles of other healthcare providers/ responders. They can coordinate and communicate with each other by unifying the approach and language among all responders. Inter-professional learning will ultimately improve the quality of care and assistance provided during a disaster.
Core disciplines related to domains	The core disciplines include three main areas: 1 Clinical and psychosocial care, which includes, but is not limited to, pre-hospital triage, tagging, treatment, and transport; emergency department triage, stabilization, care, physiological first aid, and referral to facility-based specialty coordination and psychological care. 2 Public health includes, but is not limited to, environmental health monitoring, hazardous material handling and safety, relief worker disease surveillance, disease outbreak investigation. 3 Emergency and risk management includes, but is not limited to, site security; urban search and rescue; incident command and emergency operations center management; hazardous materials management; geographic information systems; resource mobilization; public information; and media relations.
Subject areas	Subject areas can be focal or general For general, the competency set within each level extensively covers a full range of topic areas relevant to the target audience. In the focal area, the competency sets within the same level can cover a specific topic or focal area (chemical, biological, radiological and nuclear threats) that a learner may need to know.
Program duration	Short term course: range from 1 to 7 days Intermediate term course: more than 7 days, usually 2 weeks residency course and 8 weeks for field visits or online material, for undergraduates, for one academic semester.
Competency based program	The term competency highlights the set of knowledge, skills, and attitudes essential to accomplish a task successfully and professionally. The competency statements define the level of knowledge, skills, and attitudes that individuals need to demonstrate to meet the training goal. It includes an action verb describing the level of performance and a description of the subject matter, type of performance, and/or performance outcome.
Language	According to international standards, native language or a language commonly used and spoken in that country, based on the type of the course, i.e., domestic or international courses.
Modality to deliver the course	Face to face, virtual-online or combination of both.
Education and training method	Traditional (lectures) vs. blended learning.
Program accreditation	Accreditation through international organization, or internal Saudi Health Commission.
Refresher course	Refresher courses are required annually if healthcare providers are not exposed to critical events on a regular basis, as their knowledge and skills in responding to such events can be reduced.
Program evaluation	The Kirkpatrick model consist of 4 levels: A Level one: Evaluates the thoughts and feelings of trainee (feedback form) B Level two: Cognitive assessment level (skills and knowledge) (Pre & Post assessment forms) C Level three: Behavior evaluation of the outcome of the training program, this requires long term follow up & interviews with trainees D Level four: Results describe how the training affected trainees' performance in business or work environment

#### Context

The context for this review was all healthcare facilities and organizations, such as primary care centers, hospitals, Red Crescent organizations, mass gathering centers, non-government health organizations, and schools of medicine, nursing, EMS, and health sciences, in Saudi Arabia. Community establishments and rehabilitation facilities were excluded.

### Search strategies

A systematic database search was used to identify keywords related to PCC (25, 26). An initial search was conducted in 2021 on PubMed helped determine relevant medical subject heading (MeSH) keywords and phrases. These keywords and phrases (see Supplementary Table S1) were used to identify relevant articles from Public/Publisher MEDLINE (NLM Journal Articles Database) PubMed, Saudi Digital Library, The Cumulative Index to Nursing and Allied Health Literature (CINAHL), and Google Scholar. The reference lists of the fully reviewed articles were also searched to identify additional relevant articles.

### Literature identification

**Figure 1:**
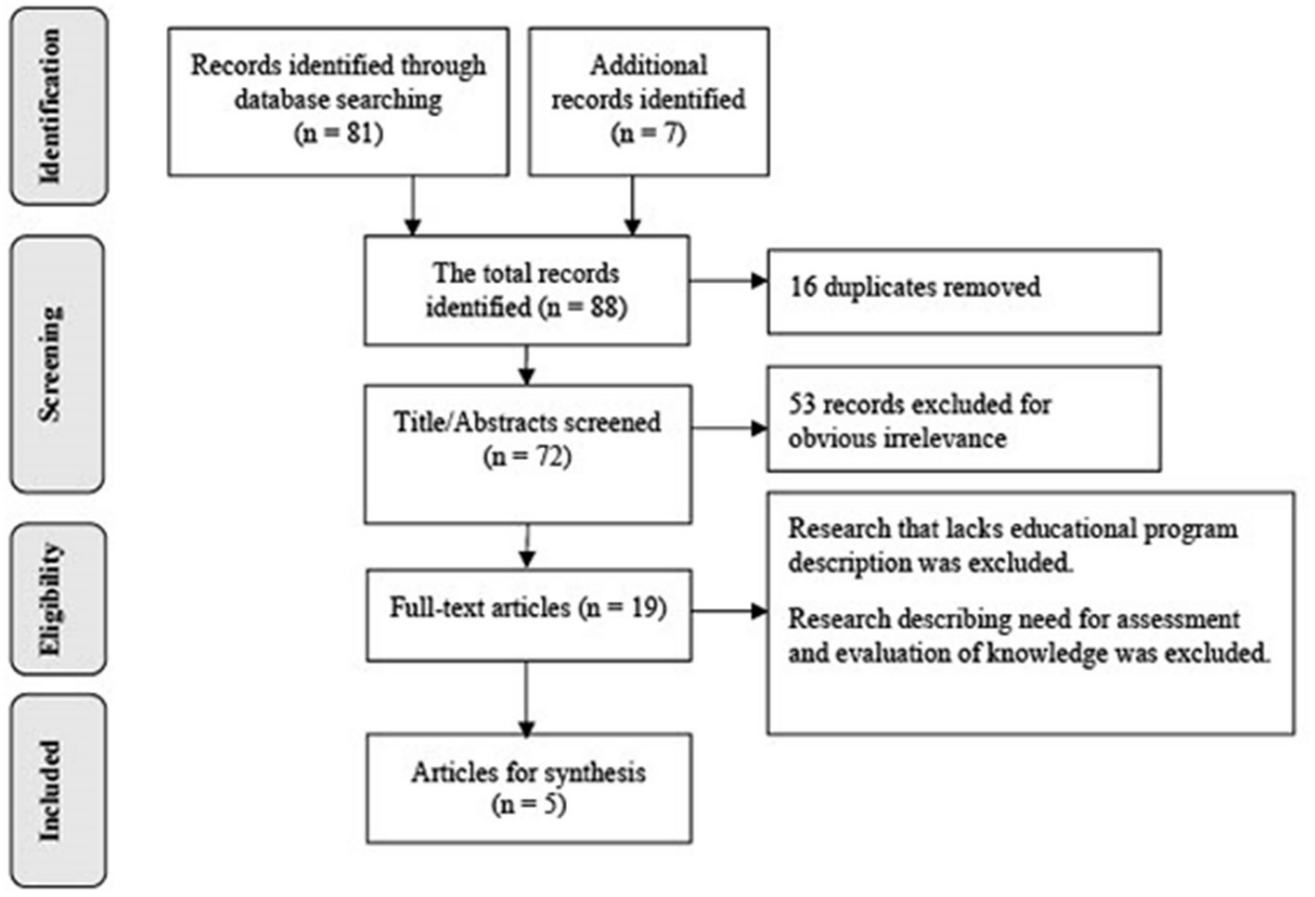
PRISMA flow diagram for the scoping review process. Adapted from Peters et al. (25).

The systematic database search resulted in 88 articles. A total of 16 duplicate articles were removed resulting in 72 articles. Titles and abstracts of the resultant articles were read in accordance with the eligibility criteria, and ineligible articles were removed. A total of 19 articles were independently read in full by all authors. Disagreement among authors was resolved by discussion. Five articles met all the eligibility criteria and were included in this review. Full details of the search strategy are provided in Figure 1.

## Results

Five articles that described disaster health education programs in Saudi Arabia were included. The relevant details from the articles including authors' names, publication year, study design, multidisciplinary program, level of training, subject area, competency-based program, domain, duration, education delivered, language delivered, accreditation, evaluation tools, teaching strategy and refresher course within 1 year are summarized in Table 3. These articles met the eligibility criteria laid out in the elements of disaster health education framework (1, 11, 23, 28–32).

**Table 3:** Articles that met the eligibility criteria according to elements of the disaster health education framework.

**No.**	**1**	**2**	**3**	**4**	**5**
Publication	Aljerian et al. (18)	Bajow et al. (19)	Bajow et al. (4)	Bajow et al. (20)	Bajow et al. (21).
Study design	Quasi-experimental	Mixed approach	Mixed approach	Qualitative	Quasi-experimental
Level of training	Foundation level	Awareness level	Awareness level	Awareness level	Awareness level
Multidisciplinary	No	Yes	Yes	Yes	Yes
Discipline	Main core disciplines	Main core disciplines	Main core disciplines	Main core disciplines	Main core disciplines
Subject area	General	General	Focal	General	General
Duration	2–4 weeks	3–5 days	3–5 days	2–4 weeks	2–4 weeks
Competency based program	Yes	Yes	Yes	Yes	Yes
Language	English	English	English	English	English
Education style	Face to face	Face to face	Face to face	Both	Both
Teaching strategy	Blended learning	Blended learning	Blended learning	Blended learning	Blended learning
Accreditation	Internal	Internal	Internal	International	International
Evaluation tools	1 and 2	All of them	All of them	None of them	All of them
Refresher course within 1 year?	No	No	No	No	No

To explore the current state of disaster health education, it is important to identify the current target audience that includes all health professionals at various levels. The awareness levels pertaining to healthcare workers for undergraduate medical students as well as healthcare providers were covered in four articles (4, 19–21). Two of the five articles were found to include undergraduate curricula and two covered healthcare providers (4, 19–21).

The two undergraduate articles were related to each other. One pertained to qualitative research with regards to a community-based disaster medicine curriculum for undergraduate medical schools. The second article included a quasi-experimental study focussed on assessing the curriculum's efficacy in terms of enhancing the knowledge of medical students in Saudi Arabia. For front-line workers (hospital receivers and responders), the foundation level was put forward with one article encompassing training for emergency department residents and staff with a primary role during disasters (18).

One article put forward advanced and intermediate levels pertaining to professional and health organizations (4). One of the articles (18) did not include a multidisciplinary/multi-sector approach with regards to disaster health response. In the articles that did promote this approach, safety and security, civil defense and police were involved for both postgraduate and undergraduate training programs (4, 19–21). One of the five articles included a program that focused on complex humanitarian emergencies (4). All five articles can be regarded as competency-based programs that included an on-site (face to face) teaching strategy, except for the community-based disaster medicine program pertaining to undergraduates. It included three live video lectures presenting the emergency medical service in Jizan, the role of the Ministry of Health and Red Crescent during population displacement in Jizan, and principles for community disaster participation and awareness. The education materials were kept on the website of the training institute for 8 weeks to help the students interact with tutors and complete their disaster community-based projects (12, 21).

Blended learning teaching methods were observed in all studies. The intermediate course duration (2 weeks) was recorded in both undergraduate and postgraduate programs (18, 20, 21). Undergraduate medical students from Jizan University received an international certificate from the Center for Research and Training in Disaster Medicine, Humanitarian and Global Health (CRIMEDIM) Novara Italy (21). English was the teaching language for all these medical sciences courses (33).

The four levels of Kirkpatrick's models for program evaluation were used to measure reaction, learning, behavior, and results in three articles (4, 19, 21). For third and fourth level assessment, the participants were approached by email and telephone 9–12 months after the course to obtain an understanding of their attitudes and experience with disaster-related topics after the course had concluded. In “Evaluation of Change in Knowledge and Attitude of Emergency Medicine Residents” (18) level one and two were used for measuring reactions and learning. None of the five studies conducted refresher courses within 12 months post-training.

### Strengthen and weakness in the five articles

The strengths and weaknesses of each of the programs described in the five articles are summarized in Table 4. The key core disciplines were the focus for all five articles, in which all the subject areas were general except for Bajow et al. where it was focal (4). The level of training included awareness, with only Aljerian et al. offering foundation level (18). All five programs described can be categorized as competency-based programs, using a blended learning approach and conducted in English. None of these programs had refresher courses within a year of completion.

**Table 4:** Strengths and weaknesses of the programs.

**Article**	**Strength**	**Weakness**
Aljerian et al. (18)	Domain-related discipline, competency- based program, face to face training delivery, with blended learning.	No multidisciplinary program and only two evaluation tools. No refresher course within 1 year.
Bajow et al. (19)	Multidisciplinary program, domain-related discipline, competency- based program, face to face delivery, blended learning and all evaluation tools.	No refresher course within 1 year.
Bajow et al. (4)	Multidisciplinary program, domain-related discipline, competency- based program, face to face delivery, blended learning and all evaluation tools.	No refresher course within 1 year.
Bajow et al. (20)	Multidisciplinary program, domain-related discipline, competency- based program, face to face delivery, blended learning.	No evaluation tools and no refresher course within 1 year.
Bajow et al. (21)	Multidisciplinary program, domain-related discipline, competency- based program, face to face delivery, blended learning and all evaluation tools.	No refresher course within 1 year.

## Discussion

According to WHO, healthcare management should be integrated with disaster risk management and prevention to prevent deaths, injuries, diseases, psychosocial problems, and other health impacts. Therefore, the disaster risk Management model should promote a combination of prevention and mitigation, preparation and planning, response and relief, and recovery (34). Preparedness is the most important phase in the process of the disaster risk management (DRM). Such preparedness covers “response planning, training and education, material supply management, development of surge capacity plan. Excellent plans and the best equipment may be of little or no benefit if the staff is not properly trained: “Preparedness without proper education and training is no preparedness”. High-quality education and training are one of the most important components of disaster health preparedness (1, 35, 36).

This is the first systematic scoping review to explore and map the current state of a disaster health education framework for short and intermediate training for medical personnel in Saudi Arabia. The initial step for standardizing disaster health training is to develop a disaster health education framework according to international standards and local context.

The national disaster health education framework may be used as a standardizing educational tool to monitor the learning process and consequently improve the quality and capability of the education program through its learning outcomes. Although standardization may lead to some inflexibility due to organizational differences, it may still ensure quality and uniformity of education curricula, facilitate international cooperation, and enable evaluation and continuous improvement (24). Effective education and training requires consensus on a set of core competencies with syllabuses based on a well-defined package of knowledge and skills (11).

### Allocation of healthcare personnel categories

Healthcare workers are required to work at varying proficiencies based on roles specific to crisis management, professional responsibilities, educational aspects, and responsibilities in a health institution (11, 28). Such a structure ensures that all healthcare personnel are adequately represented from an educational and job requirement perspective. Healthcare workers might be involved in disaster planning, management, response, and aftercare (28). Creating categories helps enhance the core competency skillset standards (28). The present scoping study considers all suggested levels (4, 18–21), however, to date the intermediate (professional-level) and advanced (organization-level) levels have only been proposed with no training actually conducted at these levels in Saudi Arabia (4). Several performance variations concerning information on bioterrorism are associated with differences in the workplace, education, working domain, and profession (15). The recommendations include having basic level core competencies for every healthcare worker before they move up to more advanced levels (foundation, intermediate, advanced level) (28).

### Competency-based education program

All five articles described competency-specific systems; such education is critical for building professionalism in disaster-specific medicine. Competency-specific curricula work on achieving goals such as uniformity of disaster health techniques and building cooperation at the international level when disasters strike (20, 23, 37).

Competency-specific systems demonstrate enhanced healthcare personnel knowledge and expertise during practical disaster response (23, 38, 39). Bloom's education taxonomy was used to implement six proficiency levels (knowledge, comprehension, application, analysis, evaluation) concerning the fundamental competencies for each of the suggested levels (4, 19–21). A previous systematic review suggested a lack of agreement concerning core competencies even for the language used across articles (11). The “competency statement” provides an appropriate description of the observable, measurable, and targeted goals achieved by learners (11, 29).

### The multidisciplinary/multisector/ interprofessional approach

Four articles assessed multidisciplinary efforts across several professions and domains (4, 19–21). Responder roles can be understood better from a multidisciplinary or inter-domain perspective; collaborating in challenging situations is critical for effective disaster handling. A multidisciplinary system is required to reduce mortality and morbidity for disaster victims and strengthen healthcare infrastructure (40, 41).

### Disaster health domain related disciplines

The World Association for Disaster and Emergency Medicine (WADEM) devised a structure for disaster-specific health information using the BRADT framework. Health aspects are part of three fundamental interrelated domains: (1) Psychological and clinical therapy, (2) Public healthcare, and (3) Risk management and emergency handling. As intersecting circles with context and support areas in proximity, support is understood as the set of disciplines, such as engineering or geography, which complement the core. The context domain comprises political and social sciences, community health, and socioeconomic study (23), but most previously recommended educational categories emphasize the three fundamental disciplines (28, 30). Moreover, each of the five articles used for this study used the three fundamental disciplines (4, 18–21).

### Education delivered

Training described in the three relevant articles was offered face to face (4, 18, 19), community medicine for the disaster scenario was also offered as distance learning (2, 20). Face to face is the prevalent teaching technique because it facilitates teacher-learner interaction (24). Healthcare personnel have time constraints concerning on-site training; hence, distance and blended learning programs are also effective for disaster medicine education (42, 43).

### Course language

The training described in the five articles was delivered in English (4, 18–21). In 2004, WADEM conducted a survey concerning the prevalent disaster health education scenario. Respondents indicated that they were trained in the native or common language used in their country. About 29% of respondents suggested that local and international scope training be separated and that native-language based teaching be prepared using translation (23).

### Blended learning

Conducting training courses with blended learning in the health care system is considered one of the important steps for disaster risk reduction management (44).

Blended learning is one of the most successful teaching methodologies in the disaster medicine field and is regarded as a critical method for students to obtain the relevant knowledge and skills (43, 45). It has been established that learners in courses that use blended learning are more certain of their knowledge for no <6 months after the training (46). Various learning strategies and methods were used in the five reviewed articles (lectures, case study, group discussion, mock simulation, experiential exercise, table-top exercise, role play and drill) (4, 18–21).

Simulation-enhanced training was the most used in reviewed articles and has been shown to be realistic and practical approach for the responders by increasing their knowledge, enhancing self-confidence, and improving their clinical skills in situations mirroring possible disaster scenarios (44, 47–50).

### Program duration

It was observed that for all healthcare awareness, basic, intermediate, and advanced levels for other global disaster health teaching frameworks, the training period ranged from 1 to 14 days, with an additional 8 weeks for field work for advanced courses (32, 33, 35–51). The education courses described in the five review articles matched these aforementioned frameworks.

### Program accreditation

All programmes included in the five articles were accredited, with just one accredited internationally (21). A 2004 WADEM survey regarding the status of disaster health training reported that most of the training programmes (65%) had been accredited with 23% locally accredited and 13% globally accredited (23).

### Subject areas

The subject area for competency sets at every education level can be either focal or general. In the case of focal subject areas, the sets of competencies may only include one subject area of particular interest without categorical specialization in that specific focal area. In an elaborate humanitarian emergency programme, the professionalization at the foundation level was limited to Community Awareness and basic tasks in the recovery stage (4, 31).

### Program evaluation

The assessment method is essential to demonstrate the program's need and to indicate how it contributed to achieving the goals and objectives of the participating individuals as well as the organizations. Good evaluation methods should be simple and adaptable (24). All phases of Kirkpatrick's Four Stage Model were stated in three articles (4, 19, 21) to develop cognitive evaluation, analysis of feedback and post-event forms for questionnaires. Competency-based exercise was used to evaluate the training goals by employing tools to assess the learning processes. Several methods can be employed, such as observation of skill demonstration, and pre- and post-program tests. Disaster health training processes differ from other types of medical education because of the wide variation in disaster patterns and the aspects that affect the response outcome. Therefore, no single approach can evaluate the value of such educational programs, and more than one approach is required to evaluate disaster health education programs (24, 29, 52–54).

### Refresher courses

The learners must retain their understanding and skills obtained in a program over time. The technical skill-based modules were observed as deteriorating at a more rapid rate than knowledge. On the basis of this finding, short refresher programmes are required every year if healthcare professionals do not get enough experience of critical disasters regularly since their skills and knowledge in handling such events can decline within 6–12 months following their preliminary training (54–56).

### Disaster health education gaps

This scoping investigation highlighted several gaps that must be considered and approached. The most common gap is that all the training programs do not contain a refresher course; another gap is that the focal subject area was introduced in just one program and this gap was stated in previous review articles which dealt with the knowledge of, and readiness of healthcare professionals in a bioterrorism situation. In this earlier review, around 43.1% of participants agreed that there was a lack of training opportunities available on bioterrorism readiness (15).

The third gap is an absence of training courses for intermediate (specialized) and advanced levels (organizational); the absence of training at specialized levels has been raised in previous studies dealing with nurse disaster education courses (13, 14, 57). Finally, the shortage of evidence-based disaster health training courses for both undergraduate and postgraduate levels was also reported in other review articles (13, 14). It was noted from previous reviews in Saudi Arabia and from the scoping review articles that each sector has different interests and there is no unified education framework (4, 13, 14, 18–21, 57).

Lack of agreement regarding the training needs of the target audience was observed in certain courses due to the manner in which the target audience was selected, such as by inviting governmental organizations where the selection of employees had no relation to their role in disaster situations. All these gaps can cause fragmentation of nationwide educational infrastructure; the same obstacles were discovered in global systems of standards, strategies, and accreditation in the domain of disaster health training and education (23).

## Limitations, implications, and recommendations

The main limitation of this study is the scarcity of peer-reviewed studies that related to the disaster health curriculum and included the elements of the disaster education framework for short and intermediate training in Saudi Arabia. Despite the limited available research, this study has several implications for improving disaster health education for short and intermediate training in Saudi Arabia. These include:

Develop competency-based multidisciplinary disaster health education courses at all educational levels, and according to the proposed disaster health framework (1, 11, 23, 28–32).

- To ensure the quality and uniformity of the education curriculum, facilitate international cooperation and enable evaluation and continuous improvement.
- Refresher programs should be available in all disaster health training programs to preserve the knowledge and skills gained from those programs.
- Encourage research publications to increase information on evidence-based disaster health programs to give opportunities to nationwide training centers to share their experiences and enhance the quality of the disaster health training programs.

To achieve these objectives, it is suggested that the Kingdom of Saudi Arabia develop an organization for disaster health education with an exclusive budget from various governmental and non-governmental organizations, managed by disaster health experts. The central aim of this organization will be to increase educational programs for disaster health education at all levels of the medical community and promote research in the field of disaster health education in Saudi Arabia.

## Conclusion

Competency-based multidisciplinary disaster health training programs are a keystone for disaster readiness. In this scoping review, it is apparent that there is a shortage of evidence-based programs for disaster health education for the short and intermediate term in Saudi Arabia. Adopting the proposed framework for disaster health education could enhance the quality and consistency of the program curriculum for health professionals, improve disaster preparedness of all sectors of the healthcare system, and meet international disaster management standards.

## Data availability statement

The original contributions presented in the study are included in the article/Supplementary material, further inquiries can be directed to the corresponding author/s.

## Author contributions

NB designed the study and drafted the manuscript, which was revised and completed by LM, NM, SA, ZM, and SA. All authors have approved the final version of the manuscript for submission.

## Conflict of interest

The authors declare that the research was conducted in the absence of any commercial or financial relationships that could be construed as a potential conflict of interest.

## Publisher's note

All claims expressed in this article are solely those of the authors and do not necessarily represent those of their affiliated organizations, or those of the publisher, the editors and the reviewers. Any product that may be evaluated in this article, or claim that may be made by its manufacturer, is not guaranteed or endorsed by the publisher.
