# Supplementary material for: Disaster health education framework for short and intermediate training in Saudi Arabia: A scoping review
Source: Front Public Health. 2022 Jul 29;10:932597. doi: 10.3389/fpubh.2022.932597 (PMC9372336; doi:10.3389/fpubh.2022.932597)
Supplement: Supplementary file 1 [file Table_1.DOCX]

**Supplementary Table S1: Concept Map**

| Population | Concepts | Context (in Saudi Arabia) |
| --- | --- | --- |
|  |  |  |
| MeSH^a^ Terms | **MeSH Terms** | **MeSH Terms** |
| - Health personnel - Undergraduates - Postgraduates - Hospital receiver | - basic level - professional level - organization level - strategy, tactical operational tracks - Multidisciplinary - Competency-based program | - Health care facilities - Organization - Hospital - Schools of health science, medicine, EMS, nursing |
| Keywords and phrases | **Keywords and phrases** | **Keywords and phrases** |
| - Health care provider - Front line provider (responder/receiver) - Disaster medicine expert - Medical dean | - Disaster medicine education - Disaster medicine curricula /curriculum - Disaster training - Core competencies - Disaster triage - Disaster health education | - Hospital - Red Crescent - Emergency Medical Service - Mass gathering |

^a^ MeSH : MEDICAL SUBJECT HEADING
